# Outcomes for older people with long-term conditions attending day care services delivered by paid staff or volunteers: a comparative study

**DOI:** 10.1177/26323524211030283

**Published:** 2021-07-09

**Authors:** Catherine Lunt, Chris Shiels, Christopher Dowrick, Mari Lloyd-Williams

**Affiliations:** Academic Palliative and Supportive Care Studies Group and Department of Primary Care and Mental Health, University of Liverpool, Liverpool, UK; Statistician, University of Liverpool, Liverpool, UK; Primary Care, Department of Primary Care and Mental Health, University of Liverpool, Liverpool, UK; Professor of Palliative Medicine, Academic Palliative and Supportive Care Studies Group and Department of Primary Care and Mental Health, University of Liverpool, Brownlow Hill, Liverpool L69 3GB, UK; Honorary Consultant Palliative Medicine, Marie Curie Hospice, Liverpool, Liverpool, UK; Liverpool CCG, Liverpool, UK; Liverpool Health Partners, Liverpool, UK

**Keywords:** Covid-19, day care services, long-term conditions, older people, rural, urban, volunteers

## Abstract

**Background:**

Day care services support older people living with long-term conditions (LTC’s).

**Aims:**

The aims of the study were to determine outcomes in terms of loneliness and health-related quality of life for older people with LTCs attending day care services in the United Kingdom.

**Methods:**

Newly referred older people with LTCs to day care services in North West of England and Wales were invited to participate. The EQ-5D-3L and De Jong Loneliness questionnaires were completed at recruitment, 6 and 12 weeks.

**Results:**

Ninty-four older people (64% female), age range 65–99 years; mean number of LTCs 4.3 (range: 2–9) were recruited. About 52% lived alone and 36% lived in one of the 20% most deprived local authorities in England and Wales. Outcomes over 12 weeks were comparable for paid, blended, and for volunteer-led services.

**Conclusion:**

Following the Covid-19 pandemic, it is increasingly urgent to support older people with LTCs who may have lost physical and cognitive function during lockdown and to support their recovery. Our study suggests that volunteers can provide services and complement the care provided by paid staff, freeing up resources and enabling increasing numbers of older people to be supported.

## Background

Globally, the number of people aged over 60 years is expected to increase by 56% by 2030.^1^ Across Europe the oldest old (over 85 years) population are projected to increase from 5.4% of the population in 2016 to 12.7% by 2030.^2,3^ Many older people live with long-term conditions (LTCs) which are lifelong incurable conditions requiring drugs or treatment for symptom management,^2,3^ and which can lead to increasing social isolation and reduction in physical and mental health. In the United Kingdom, people with multiple LTCs, for example, heart disease, diabetes, respiratory disease, are high users of health services^4,5^ and multiple LTCs result in higher healthcare costs^6^ and more than a third of people aged over 75 in the United Kingdom take four or more medicines.^7^

Older people are more likely to experience loneliness due to bereavement, declining health, or decreased independence.^8^ In the United Kingdom, loneliness has been highlighted as a public health issue^9^ for all age groups. The term loneliness is often used interchangeably with that of social isolation; however, loneliness can be experienced by those with social networks. Therefore, loneliness is considered to be a mismatch between the person’s desire or expectation in the number and quality of connections with others and the actual connections in their day-to-day lives. Weiss^10^ suggests that loneliness has social and emotional dimensions and is categorised as unpleasant and unchosen, dominated by feelings of disconnection, confinement, and fears of dependency.^11^ An integrative review^12^ investigating interventions reported features of services which successfully reduced loneliness to include the adaptability of the service, community development approaches, and productive engagement.

A definition of day care services is

A day care service offers communal care, with paid or voluntary carers, in a setting outside the user’s home. Individuals come or are brought to use the services, which are available for at least four hours during the day, and return home on the same day.^13^

Day care services can support older people living at home with multiple LTCs, to age in place and to live independently.^14–17^ Day care services support older people by giving an opportunity for them to socialise, meet others, and thereby reduce loneliness.^18^ Activities can include crafts, gardening, baking, quizzes, and memory games along with activities such as chair-based exercises, exercises to improve balance, and Tai Chi, all with the aim of promoting physical and cognitive function,^19^ and attendance is usually not time limited. Day care is often discussed in the literature with regards to the respite provided for carers rather than any outcomes and benefits that attendance at day care services may have on older people themselves and indeed few day care services routinely use outcome measures.

Amid the backdrop of austerity in the United Kingdom, adult social care funding has reduced 17% since 2009/10.^20^ In response, many local authorities have increased user fees or co-payments for care services. Older people at greatest risk of loneliness are those with less financial resources, living in socially deprived areas and lacking access to care or social activities, and age is associated with an increased chance of exclusion.^21^ In a paper examining the relationships between neighbourhood characteristics, personal attributes and level of social exclusion in later life,^22^ it was reported that ageing in place and stronger attachments to neighbourhood were associated with lower levels of social exclusion. The COVID-19 pandemic and strategies to shield people with LTCs^23^ has highlighted the role non-health organisations play in supporting people who are isolated to maintain their well-being, with many relying partly or solely on volunteers. The Caring for our Future White Paper^24^ enables local authorities to relinquish the delivery of adult day care services to private, public, or voluntary sector organisations. The configuration of the services is varied, and care may be delivered by either a paid workforce, volunteers, or a combination of both.

A lack of a standardised definitions of day care services makes determining effectiveness challenging^19^ to understand what works, for whom and in what circumstances within day care settings,^25^ and little is known about those attending day care and any outcomes or benefits for day care users.^16,26^ A recent paper published in the United States after the completion of our study^27^ has aimed to develop consensus outcomes for day services and focussed on three areas of participant and carer well-being and healthcare utilisation; however, the views of those attending Day Care Services and their carers did not appear to be included within the process of developing outcomes.

The aim of this study was to determine outcomes in terms of loneliness and health-related quality of life of day care attendance for older people with multiple LTCs attending services provided by paid staff (local authority and independent/private day care centres), voluntary services (delivered entirely by volunteers) and blended services (a small number of paid staff supported by volunteers) and to examine any differences in outcomes by service type.

## Methods

### Settings and recruitment

This study was carried out in North West of England and Wales with nine generic older day care services who, while all accepting patients with dementia, were not a specialist day care services for people with dementia or any other condition; two centres employed paid staff only; five were a blended service of a small number of paid staff with a number of volunteers and two were managed and run entirely by volunteers. Six of the nine centres were located in the highest two deciles of areas of multiple deprivation, including both paid services, three of the five blended services and one of the two voluntary services; however, all nine services included areas of significant multiple deprivation within their local areas. All services accepted referrals from health and social care workers and from families and accepted self-referrals. All of the services provided a similar range of activities with blended and volunteer-led centres appearing to offer greater diversity of activities then paid services. The aims of the services involved in the study were to support older people, improve quality of life and to help older people engage/re-engage with their communities making new social contacts with aim of reducing social isolation and loneliness. For older people meeting ‘eligibility thresholds/criteria’ indicating more complex needs, local authorities fund Day Care places within local authority provided day care services or fully fund places within a private day care provider. All services included in the study were referred older people who had received a needs assessment exploring physical, cognitive, and social well-being; however, due to the presence of specialised equipment, for example, hoists only paid services were able to support older people with very complex needs and who required hoisting and greater assistance. Inclusion criteria for the study were older people aged 65 years and older, more than one LTC, living at home, able to give informed consent and an expected prognosis of at least 3 months. Exclusion criteria were cognitive impairment (assessed by Day Centre Managers/Leaders) which would limit the older person being able to give informed consent and complete questionnaires; unable to understand written/spoken English and an estimated prognosis of less than 3 months. Day centre managers/leaders were invited to inform all eligible new referrals regarding the study and to provide written details of what the study entailed with those interested invited to contact the researcher. All participants were provided with a patient information sheet explaining the purpose of the study and gave written consent to participate in the study. The majority of baseline interviews at recruitment were conducted at the day centre with some at the participant’s home if there was insufficient time at the day centre to allow the baseline data to be collected. Full ethical approval was obtained (Research Ethics committee 000967). Recruitment into the study occurred during 2016–2017.

At recruitment, baseline information included age, gender, ethnicity, marital status, residential status, carer status, number and type of LTCs using the Charlson Morbidity Index^28^ as a method of identifying LTCs, EQ-5D-3L and the De Jong Loneliness 6 item questionnaire. The EQ-5D-3L and the De Jong Loneliness 6-item questionnaire were administered at recruitment/baseline, and at 6-week and 12-week follow-up. Participants usually completed the follow-up questionnaires by post with a small number opting for researcher contact which was usually by telephone. Due to the vulnerability and frailty of the sample, at each time point, the service was contacted to determine if it was appropriate to contact each participant prior to contacting for follow-up. All data collection was paper based.

### Measures used

The EQ-5D-3L is widely used to measure health-related quality of life and is validated for older people.^29^ The 5-item questionnaire includes following domains: mobility; self care; usual activities; pain/discomfort and anxiety/depression^30^ and a visual analogue scale. Each domain has three levels of response–no problems, some/moderate problems, and extreme problems.

The De Jong Giervald Loneliness Scale is a 6-item measure and does not use the term loneliness to avoid any associated stigma. The scale addresses Social and Emotional Loneliness with Social Loneliness associated with reduced social networks and individual resources, and emotional loneliness relating to the absence of intimate relationships such as partner or close other.^31^ A total score of 0 means that there is no evidence of loneliness and score of 6 indicating intense loneliness.

The Charlson Morbidity Index^28^ was utilised to capture the number and types of LTCs.

### Statistical analysis

Univariate analysis was conducted in order to describe differences in the baseline characteristics of the groups of clients using a particular type of day care service (paid, blended, and voluntary). The significance of association between baseline attributes/outcome scores and membership of service type and location groups was tested by the chi-square test for categorical variables and the *t*-test/one-way analysis of variance (ANOVA) for continuous measures.

Differences in mean scores between the client groups at each time point (baseline, 6 weeks and 12 weeks) were investigated using the *t*-test or a one-way ANOVA. Repeated-measures two-way ANOVA was used to test for between-group differences in changing scores over time.

Univariate logistic models were run in order to estimate the effect of type and location of service on the likelihood of ‘any improvement’ in outcome (a reduction in loneliness score, decrease in number of reported EQ5 problems, increase in VAS global health rating) from baseline to final follow-up. Odds ratios, 95% confidence intervals, and associated *p* values are reported.

For all analyses, a conventional criterion of statistical significance (*p* < 0.05) was used.

All data were analysed using SPSS for Windows 22.0. We analysed all the data available, and we did not carry out imputation for missing data.

## Results

### Composition of the group of service-users

**Figure 1.**
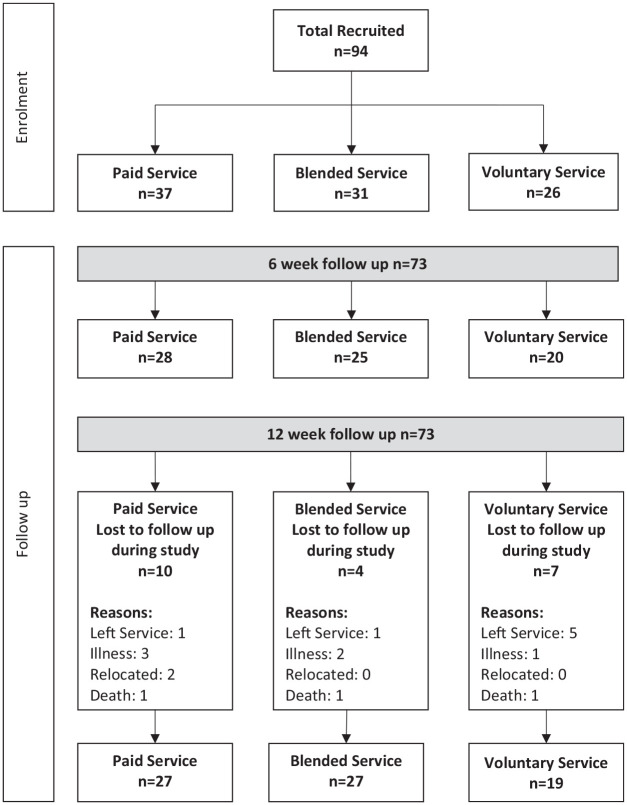
Flow chart.

Ninety-four participants (64%female), age range 65–99 years (mean age 82 years) from nine centres were recruited to the study, and completed baseline measures (Figure 1) and Table 1 provide a description of each day care centre. All those who the day care service manager/leader believed were eligible and wished to contact the researcher agreed to participate. The number of LTCs ranged from 2 to 9 (mean of 4.3 LTCs). The most commonly reported LTCs were arthritis, heart disease, early to moderate dementia, stroke and mental health issues. The vast majority attended day services on 1 day of the week, with a small number attending 2 or more days. Thirty-two percent were married, 56% widowed and 12% separated, divorced or never married, and 52% of participants lived alone. Over a third (37%) identified a carer who was a family member living with them, and 27% identified a family member as a carer living elsewhere. More than a third (36%) of those recruited lived in one of the 20% most-deprived local authorities in England and Wales. On average, older people travelled 3 miles to attend the day services, with majority of participants across all centres utilising disabled transport provided by the centre or transport arranged by relatives and small number transported by family or friends (range: 0.1–20 miles in services located in more rural areas). Five centres served both rural and urban areas, and one centre served a largely rural area – the distance travelled to services related to where participants lived and in all the services serving rural areas, these included areas of significant rural deprivation. About 73 participants (78%) completed follow-up at 6 weeks and 12 weeks.

**Table 1.** Description of day care settings.

Name	Service type	Provider	Operational times/cost	Accept participants from urban/rural	Facilities	Activities	Index of multiple deprivation	Deciles of deprivation (1 being highest area deprivation)
Sunflower	Paid	Statutory	7 days per week 9 am to 4 pm £94 per week 15 places per day	Urban	Purpose built centre with adjoining areas linked to re-ablement service with inpatient beds. Day centre room within a suite of shared rooms (large multipurpose room, small consulting room and large therapies room with exercise equipment). Service provider owns building. Lunch takes place in adjoining area provided by same organisation or participants eat out (lunch additional cost to participants).	Quiz, cognitive games, arts and crafts, music excursions, visits from community groups.	29	1
Snowdrop	Paid	Independent	5 days per week 9:30 am to 5 pm £25 per day 15 places per day	Urban	Purpose built community/day service with adjoining large hall used for community groups, a therapy room, a café and craft lounge. The centre is run by the independent company that also runs day care. Service provider owns building. Lunch takes places in the main hall where the day care attendees eat with just over 50 people attending only for lunch as part of a lunch club. Lunch provided by centre	Quiz, cognitive games, chair-based exercise, arts & crafts, dance & movement sessions.	1355	1
Beech	Blended	National charity	4 days per week 10 am to 3:30 pm £16 per day 20 places per day	Urban and rural	Day care take place in a lounge in a multifunctional centre with a multipurpose room for exercises and a small room for hairdressing. Lunch takes place in a large hall, where day care attendees eat with people attending for lunch only as part of a lunch club. There is a kitchen specifically for the day centre and lunch club. The centre also has a drop in café for the wider community and a church. The centre that runs the day care manages the building. Cost of lunch included in price for day care.	Cognitive games, quiz, music, craft, chair-based exercise	1331	1
Birch A	Blended	National charity	2 days per week, 10 am to 2:30 pm £12.50 per day 15 places per day	Urban and rural	Day care takes place in a large multipurpose room within community housing accommodation. Food is provided in a café/bistro. The facilities and food are not provided by the same charity that provides day care. Lunch price included in price for day care.	Quiz, cognitive games, board games, chair-based exercise, singing, arts and crafts, visits from community groups and talks given local groups.	6304	2
Birch B	Blended	National charity	2 days per week, 10 am to 2:30 pm £12.50 per day 15 places per day	Urban and rural	Day care takes place in a large multipurpose room within community housing accommodation. Food is provided in a café/bistro. The facilities and food are not provided by the same charity that provides day care. Lunch price included in price for day care.	Quiz, cognitive games, board games, chair-based exercise, singing, arts & crafts, visits from community groups, talks given local groups.	28,845	2
Blackthorn	Blended	National charity	2 days per week, 10 am to 2:30pm £25 per day 25 places per day	Urban and rural	Day care take places in two rooms rented in a social club with participants given access to the snooker room. The charity that provides the day care does not own the facilities or provide and prepare the food. Cost of lunch included in the price of day care.	Quiz, cognitive games, board games, chair-based exercise, arts & crafts, music, singing.	19,098	6
Ash	Blended	Regional charity	1 day per week 10 am to 2:30 pm £15 per day 15 places per day	Urban and rural	Day care takes place in a room within a multi-use community centre, with other groups using the rooms on other days. The centre does not own the buildings it uses however the charity does prepare and supply the meals that are delivered each day, ready made by the charity. Lunch is included in the price of day care.	Quiz, cognitive games, board games, chair-based exercise, singing, dancing, arts & crafts.	8813	3
Lily	Voluntary	Local voluntary group	4 days per week, £3.70 per day 9 am to 2pm 30 places per day	Urban	Day care takes place in a large hall within a community centre with a kitchen adjacent to the room. The day care volunteers prepare and serve the food. The service does not own the centre and pays rent to the local council. The cost of lunch is included in the day care service.	Quiz, cognitive games, board games, chair-based exercise, singing, arts & crafts, visits from community groups.	3994	2
Poppy	Voluntary	Local voluntary group	2 days weekly & 1 fortnightly - £8/day 10 am to 3:30 pm 15 places per day	Rural	Day care takes place in a small number of rooms adjacent to a Chapel. Food is prepared on site by a Paid cook. There is a dining room, a small lounge area, conservatory/reception area and an activity room. Lunch included in day care price	Chair-based exercise, singing, art & crafts, cognitive games, talks by local people literature, poetry, local history, painting	1340	8

### Baseline characteristics of service-users, and type and location of service

Table 2 reports the demographic profile of the older people attending day care services delivered by paid staff, paid and voluntary staff (‘blended’) and voluntary staff only.

**Table 2.** Baseline characteristics and outcome scores of clients using different types of service.

*Column percentages*	Type of service			P
	Paid	Blended	Voluntary	
	% (n/N)	% (n/N)	% (n/N)	
Gender				
Male	35 (13/37)	29 (9/31)	46 (12/26)	0.40
Female	65 (24/37)	71 (22/31)	54 (14/26)	
Age group				
Mean age	80.9	84.7	80.4	0.04
Marital status				
Currently married	27 (10/37)	22 (7/31)	50 (13/26)	0.11
Separated or divorced	16 (6/37)	10 (3/31)	0	
Widowed	54 (20/37)	68 (21/31)	46 (12/26)	
Never married	3 (1/37)	0	4 (1/26)	
Social deprivation				
Living in one of 20% most deprived LSOAs in Eng or Wales	56 (20/36)	24 (7/29)	30 (7/23)	0.02
Mean distance between home and centre	2.0	2.11	5.84	0.001
Living arrangements				
Partner present no children	16 (6/37)	23 (7/31)	38 (10/26)	0.22
Children are present but no partner	19 (7/37)	19 (6/31)	12 (3/26)	
Partner and children are present	8 (3/37)	0	12 (3/26)	
I live alone	57 (21/37)	58 (18/31)	38 (10/26)	
Carer status				
I have a carer who is a family member that lives with me	41 (15/37)	29 (9/31)	39 (10/26)	0.25
I have a carer who lives with me but is not a family member	3 (1/37)	0	0	
I have a carer who is a family member that does not live with me	27 (10/37)	32 (10/31)	8 (2/26)	
I have a carer who is not a family member and does not live with me	0	3 (1/31)	8 (2/26)	
I do not have a carer	30 (11/37)	36 (11/31)	46 (12/26)	
Educational status				
I hold no educational or vocational qualifications	64 (23/36)	36 (11/31)	58 (15/26)	0.13
I have educational or vocational qualifications but not a University degree	33 (12/36)	54 (17/31)	31 (8/26)	
I hold a university degree or above	3 (1/36)	10 (3/31)	11 (3/26)	
Long-term conditions				
Mean no of LTCs reported	4.4	4.0	4.7	0.39
Sensory loss – sight	62 (23/37)	74 (23/31)	50 (13/26)	0.17
Sensory loss – hearing	28 (10/36)	36 (11/31)	39 (10/26)	0.65
EQ-5D-3L				
Reported problem with mobility	76 (28/37)	81 (25/31)	73 (19/26)	0.79
Reported problem with self-care	30 (11/37)	23 (7/31)	42 (11/26)	0.27
Reported problem with usual activities	70 (26/37)	71 (22/31)	69 (18/26)	0.99
Reported problem with pain/discomfort	41 (15/37)	52 (16/31)	54 (14/26)	0.51
Reported problem with anxiety or depression	49 (18/37)	33 (10/30)	50 (13/26)	0.35
Mean VAS score	68	66	72	0.22
Mean number of EQ5 problems	2.6	3.1	2.7	0.22
De Jong SL sub-scale: reporting ‘more or less’ or ‘no’. . . .				
There are plenty of people I can rely on when I have problems	35 (13/37)	13 (4/30)	40 (10/25)	0.04
Mean EL score	1.2	1.3	1.1	0.77
Mean SL score	0.78	0.42	0.88	0.21
Mean overall loneliness score	2.0	1.7	2.0	0.68

Blended service participants were significantly older (mean age 84.7 vs 80.6, *p* = 0.04). All participants described their ethnicity as White. A significantly higher proportion of the paid service group lived in one of the most socially deprived neighbourhoods 56% compared to 27% of participants attending other services (*p* = 0.02). Those attending voluntary services had a significantly greater distance to travel (mean 5.8 vs 2.2 miles) (*p* = 0.001). A significantly lower proportion of those attending blended day care services responded positively to the De Jong item relating to having ‘plenty of people to rely on when having problems’ (13% compared to 37% of all other participants, *p* = 0.04). The number of LTCs reported at baseline was comparable across all service types (paid staff mean 4.4, blended 4.0, voluntary 4.7, *p* = 0.39).

### Service type, location and change in EQ-5D-3L self-reported health status

The proportions of older people of the three service types (paid, blended and voluntary) reporting individual health problems on the EQ-5D-3L, along with mean number of problems and VAS score, are reported in Table 3. A significantly higher proportion of participants attending paid day care services reported a self-care problem at 6-week follow-up (46%, compared to 32% of blended service clients and only 10% of voluntary service users, *p* = 0.02). However, there were no other statistically significant differences between the clients attending different services, at any time point or in the change in proportions/scores over time in terms of self-reported health status.

**Table 3.** Paid, blended and voluntary service users reporting individual EQ-5D-3L problems at baseline and follow-up.

	Baseline (P = 37, B = 31, V = 26)	6 week (P = 28, B = 25, V = 20)	12 week (P = 27, B = 27, V = 19)	*p*
	%	%	%	
Mobility				
Paid	75.7	75.0	77.8	0.79
Blended	80.6	76.0	85.2	
Voluntary	73.1	65.0	68.4	
*p*	0.79	0.67	0.40	
Self-care				
Paid	29.7	46.4	40.7	0.57
Blended	22.6	32.0	29.6	
Voluntary	42.3	10.0	26.3	
*p*	0.27	0.02	0.53	
Usual activities				
Paid	70.3	75.0	85.2	0.90
Blended	71.0	80.0	74.1	
Voluntary	69.2	60.0	73.7	
*p*	0.99	0.31	0.53	
Pain/discomfort				
Paid	40.5	46.4	44.4	0.66
Blended	51.6	48.0	48.1	
Voluntary	53.8	50.0	68.4	
*p*	0.51	0.97	0.24	
Anxiety/depression				
Paid	48.6	46.4	33.3	0.32
Blended	33.3	40.0	37.0	
Voluntary	50.0	40.0	15.8	
*p*	0.35	0.86	0.27	
Mean no of EQ-5D-3L problems				
Paid	2.6	2.9	2.8	0.75
Blended	2.6	2.8	2.7	
Voluntary	2.9	2.3	2.5	
*p*	0.73	0.27	0.80	
Mean VAS score				
Paid	68.3	71.7	74.2	0.65
Blended	66.3	74.1	71.9	
Voluntary	72.3	75.8	76.8	
*p*	0.55	0.72	0.68	

VAS: Visual Analogue Scale

When comparing services, the mean number of problems reported by older people attending voluntary day care services declined between baseline and 12 weeks, while those attending blended and paid services increased. For the domains of mobility and self-care, the proportion of participants reporting problems on these domains declined in those attending voluntary services but increased at blended and paid services between baseline and 12 weeks.

Anxiety and depression domains revealed a decline in both paid and voluntary services between baseline and 12 weeks. There was a small increase in anxiety and depression levels at blended services between baseline and 12 weeks. Pain increased between baseline and 12 weeks in paid and voluntary services. The mean VAS score for all services reported positive change in health and well-being from baseline to 12 weeks.

### Service type, location, and change in reported loneliness

There were no statistical significant differences reported for loneliness (Table 4). However, there was a trend for change in mean total loneliness between baseline and 12 weeks to reduce in blended services and Voluntary services but to increase in those attending paid staff services. In order to examine this further, the mean scores for emotional loneliness and social loneliness were compared by service group. When social loneliness group means across the three services were analysed from baseline to 12 weeks, it could be seen that the group mean score reduced across all services and the apparent absence of reduced loneliness for those attending paid staff services appeared to be connected to levels of emotional loneliness rather than social loneliness.

**Table 4.** Baseline and follow-up De Jong loneliness (total, emotional and social) scores for paid, blended and voluntary service-users.

	Total loneliness score at baseline (P = 37; B = 31; V = 26)	Total loneliness score at 6 weeks (P = 27; B = 25; V = 19)	Total loneliness score at 12 weeks (P = 25; B = 28; V = 18)	*p*
	Mean	Mean	Mean	
Paid	2.03	2.11	2.80	0.72
Blended	1.71	1.24	1.29	
Voluntary	2.00	1.79	1.65	
*p*	0.68	0.13	0.15	
	EL score at baseline (P = 37; B = 31; V = 26)	EL score at 6 weeks (P = 27; B = 25; V = 19)	EL score at 12 weeks (P = 25; B = 28; V = 18)	*p*
	Mean	Mean	Mean	
Paid	1.24	1.44	1.48	0.47
Blended	1.29	0.96	1.07	
Voluntary	1.12	1.16	0.94	
*p*	0.77	0.22	0.22	
	SL score at baseline (P = 37; B = 31; V = 26)	SL score at 6 weeks (P = 28; B = 25; V = 19)	SL score at 12 weeks (P = 25; B = 28; V = 18)	*p*
	Mean	Mean	Mean	
Paid	0.78	0.64	0.52	0.91
Blended	0.42	0.28	0.21	
Voluntary	0.88	0.74	0.44	
*p*	0.21	0.26	0.32	

EL: Emotional Loneliness; SL: Social Loneliness

### Likelihood of ‘any improvement’ in outcome

Table 5 illustrates the likelihood of improved outcomes for people attending blended services or voluntary services when compared with paid staff services. Older people attending a voluntary service were over twice as likely to experience a reduction in De Jong loneliness score between baseline and their final follow-up. Older people attending a ‘blended’ service had a raised likelihood of experiencing a reduction in the number of reported EQ5 health problems. The voluntary service group had a statistically significant increase in the likelihood of reporting fewer health problems over follow-up. In terms of reporting an improvement in the global health rating (VAS) from baseline, those attending voluntary services had a reduced likelihood however, users of blended services had raised odds of reporting a higher VAS rating.

**Table 5.** Likelihood of ‘any improvement’ in outcome between paid staff services and services with volunteers.

Service compared with paid staff services	Outcome	Odds ratio	Confidence interval at 95%	*p* value
Blended service	Reduction in loneliness score	2.01	0.65–6.22	0.23
	Reduction in number of EQ-5D-3L health problems	1.46	0.5–4.24	0.48
	Reporting increase in EQ-5D-3L VAS score	2.0	0.64–6.29	0.24
Voluntary service	Reduction in loneliness score	2.46	0.74–8.26	0.14
	Reduction in number of EQ-5D-3L health problems	3.45	1.01–12.8	0.04
	Reporting increase in EQ-5D-3L–VAS score	0.67	0.21–2.17	0.50

VAS: Visual Analogue Scale

## Discussion

Descriptors of people attending day care services are rarely reported^26,32^ and neither are service outcomes.^33^ To our knowledge, this is the first study to aim to determine outcomes of day care attendance in terms of loneliness and health-related quality of life longitudinally across different service types. Our findings suggest that older people with long-term conditions can benefit in terms of improved outcomes in loneliness and health-related quality of life from attending day centres in the first 12 weeks following referral.

There is a correlation between multi-morbidity and greater functional impairment resulting in dependence.^34^ Participants from paid staff services had met an eligibility threshold/criteria assessment in order to attend, and it would be expected that the number of LTCs would be higher than for blended or volunteer-led services; however, the mean number of LTCs reported by participants was similar across all service types as was self-reported frequency and mean number of problems on EQ-5D-3 L At paid staff services the most common LTCs reported included early stage dementia, and stroke, compared to voluntary services where diabetes and gastric conditions were the most common. This suggested that older people attending paid staff services may have met the ‘eligibility thresholds/criteria’ to attend due to personal care needs associated with their conditions, for example, stroke and early-to-moderate dementia. All day care services involved in the study accepted referrals for older people who had received a needs assessment exploring physical, cognitive and social well-being.

The proportion of people living in deprived areas was highest among those participants attending paid staff services (*p* = 0.02). Evidence from longitudinal research in the United Kingdom has established that those aged 80 years and above, with poor self-rated health predicted higher levels of exclusion, and older people living in the most deprived neighbourhoods had the highest levels of social exclusion.^22^ It is also known that general health outcomes are worse for people living in more deprived neighbourhoods.^6^ However, again it is interesting to note that, at baseline, there was no difference in self-reported health by participants accessing paid, Blended or volunteer run services. Baseline data also provided insights into issues regarding access for older people using day care services. The distance travelled varied significantly between services with those attending Voluntary services travelling further than those attending Paid staff and Blended services travelled the greatest distance (*p* = 0.001). Both paid services were located in urban areas as was one of the two voluntary services and five day centres from where recruitment took place were located in urban and rural and one volunteer-led centre was in a rural area. All of the urban and rural and rural centres had areas of significant rural deprivation within their catchment areas and all provided accessible disabled transport to allow older people from rural areas to attend.

Our study suggests that outcomes in terms of loneliness and health-related quality of life for older people attending day care services were positive regardless of whether this provision was delivered by a paid service, blended model or a service delivered by volunteers, which is similar to the findings of Orellana and colleagues^32^ who conducted a mixed-method study of quantitative and qualitative data collected at a single time point with 23 older people attending four day care services within the South of England with two centres being run by voluntary/charitable organisations (i.e., blended services) and two by paid services namely a local authority and a housing association.

Loneliness is a mismatch between the quantity and quality of a person’s relationship’s and their desire or expectation for relationships.^35^ Loneliness consists of two elements: social and emotional loneliness.^10^ Day care provides an opportunity for people to socialise and to re-engage with their community when this has not been possible due to declining health. Our study reveals a trend for a reduction in loneliness during the first 12 weeks of attending day care services. In a paper reporting on a sub-set analysis of 13 older people living with adult children and attending a re-ablement programme,^36^ found that emotional loneliness was significantly higher in this group at the start of attendance but not at the end of the programme and suggested that social groups may be effective in helping reduce emotional loneliness. Loneliness as a consequence of poor social environment can have a strong negative impact on well-being.^37^ A previous review of effective interventions to reduce loneliness revealed the components of successful interventions included adaptability, community development approaches, and productive engagement.^12^ In the blended and volunteer run services, with possibly less time being devoted to physical care/toileting there may have been more time to engage with those attending and to build close relationships. In addition volunteers usually volunteer within local services therefore in those services utilising volunteers, there may be greater feeling of community and belonging among volunteers and those attending their ‘local’ day care service. This may explain the finding that older people attending blended and voluntary services were over twice as likely to experience a reduction in De Jong loneliness score from baseline and their final follow-up.

In areas where blended and volunteer lead services were located, the numbers of Black Asian and Minority Ethnic groups (BAME) living in the area was low. However, for paid staff services the BAME population was close to the national average, but older people from BAME groups were absent from services and therefore absent from the study. This raises the question whether the lack any BAME clients reveals a barrier for people accessing and being referred to services. The assumption that older people from BAME have stronger support networks and may not want to access such support services is unfounded.^29^ It has been suggested that there is a failure in many services to market themselves effectively to people from ethnic minority backgrounds.^16^

### Strengths and limitations of the study

To our knowledge this longitudinal study is the first to attempt to investigate outcomes in terms of loneliness and health-related quality of life for older people who have multiple LTCs attending day care services by comparing outcomes across service types and provides a unique insight into the populations utilising different services types in urban and rural areas. Our findings revealed that older people attending day care services provided by blended and voluntary services reported comparable numbers of LTCs as those attending paid staff services, but the impact of the LTCs on physical and emotional function may not have been elicited fully by the measures used. Equally, self-reported frequency and mean number of problems on EQ-5D-3L was similar across all services types. Day care service managers/leaders provided new referrals with information regarding the study and we did not have ethical approval to collect any data on those who declined to receive information nor did we have ethical approval to contact those who discontinued attending day care services and have no knowledge whether their experiences of day care services were different from those who continued to attend. In this hard to reach and under-researched population, we recruited nearly 100 people and retained 78% over the 12-week follow-up. Previous studies with this population group have achieved lower recruitment and higher attrition rates.^38^ We considered it important to use all available data in our analysis but acknowledge that there could have been a skew in follow-up findings due to attrition although our attrition was low at 22%.

## Conclusion

The findings of this study addresses some of the gaps in current knowledge regarding day care service provision with regards to the nature of LTCs in older people attending day care services and subsequent outcomes in terms of loneliness and health-related quality of life within different day care service models. Our findings are important and are very relevant particularly post Covid-19 when it is known that for many older people physical, emotional and cognitive function have been severely compromised during lockdown, and loneliness has significantly increased. Covid-19 has seen a huge increase in people volunteering within their local communities and in supporting older people with errands but door-step visits and in many cases building close relationships and friendships. As the work patterns of many people change post-covid, there may be more people willing to volunteer in providing services for older people within their communities. At a time of increasing austerity within the United Kingdom, and continuing closure of paid services especially by local authorities, the development and expansion of volunteer-led and blended day care services could help provide sustainable services with improved outcomes for increasing numbers of older people with LTCs living within our communities allowing paid day care services to focus on those with the greatest physical and greatest cognitive needs.

## References

[1] United Nations. World population ageing 2019, https://www.un.org/en/development/desa/population/publications/pdf/ageing/WorldPopulationAgeing2019-Highlights.pdf (accessed 23 August 2020).

[2] Eurostat. Population structure and ageing, http://ec.europa.eu/eurostat/statistics-explained/index.php/Population_structure_and_ageing (2017, accessed 7 June 2019).

[3] Goodwin N Curry N Naylor C , et al. Managing people with long term conditions. London: King’s Fund, 2010.

[4] Foster A . Is frequent attendance in primary care disease-specific? Fam Pract 2006; 23: 444–452. 16675502 10.1093/fampra/cml019

[5] Lamb S . Permanent personhood or meaningful decline? Toward a critical anthropology of successful aging. J Aging Stud 2014; 29: 41–52. 24655672 10.1016/j.jaging.2013.12.006

[6] Barnett K Mercer SW Norbury M , et al. Epidemiology of multimorbidity and implications for health care, research and medical education: a cross-sectional study. Lancet 2012; 380: 37–43. 22579043 10.1016/S0140-6736(12)60240-2

[7] Barnett N Oboh L Smith K . Patient-centred management of polypharmacy: a process for practice. Eur J Hosp Pharm 2016; 23: 113–117. 31156827 10.1136/ejhpharm-2015-000762 PMC6451546

[8] Hagan R . Loneliness, older people and a proposed social work response. J Soc Work. Epub ahead of print 2 June 2020. DOI: 10.1177/1468017320927630.

[9] Collins AB Wrigley J . Can a neighbourhood approach to loneliness contribute to people’s wellbeing? London: Joseph Rowntree Foundation, 2014.

[10] Weiss R. S. (1973). Loneliness: The experience of emotional and social isolation. The MIT Press.

[11] Kitzmuller G Clancy A Vaismoradi M , et al. Trapped in an empty waiting room, the existential human core of loneliness in old age – a meta-synthesis. Qual Health Res 2018; 28: 213–230. 29235943 10.1177/1049732317735079

[12] Gardiner C Geldenhuys G Gott M . Interventions to reduce social isolation and loneliness among older people: an integrative review. Health Soc Care Community 2018; 26: 147–157. 27413007 10.1111/hsc.12367

[13] Tester S . Day services for older people. In: Clark C (ed.) Adult day services and social inclusion – better days. London: Jessica Kingsley Publishers, 2001, pp. 19–45.

[14] Clark C . The transformation of day care. In: Clark C (ed.) Adult day services and social inclusion – better days. London: Jessica Kingsley Publishers, 2001, pp. 9–18.

[15] Lecovich E Biderman A . Attendance in adult day care centers and its relation to loneliness among frail older adults. Int Psychogeriatr 2012; 24: 439–448. 21996017 10.1017/S1041610211001840

[16] Manthorpe J Moriarty J . Examining day centre provision for older people in the UK using the Equality Act 2010: findings of a scoping review. Health Soc Care Community 2014; 22: 352–360. 23952653 10.1111/hsc.12065

[17] Schmitt EM Sands LP Weiss S , et al. Adult day health center participation and health related quality of life. Gerontologist 2010; 50: 531–540. 20106933 10.1093/geront/gnp172 PMC3119386

[18] Aday R Kehoe G Farney L . Impact of senior center friendships on aging women who live alone. J Women Aging 2006; 18: 57–73. 16635950 10.1300/J074v18n01_05

[19] Fields NL Anderson KA Dabelko-Schoeny H , et al. The effectiveness of adult day services for older adults: a review of the literature from 2000-2011. J Appl Gerontol 2014; 33: 130–163. 24652952 10.1177/0733464812443308

[20] Quilter-Pinner H Snelling C . Saving social care. A fair funding settlement for the future. London: Institute for Public Policy Research, 2017.

[21] Kneale D . Is social exclusion still important for older people? London: International Longevity Centre, 2012.

[22] Prattley J Buffel T Marshall A , et al. Area effects on the level and development of social exclusion in later life. Soc Sci Med 2020; 246: 112722. 31972379 10.1016/j.socscimed.2019.112722

[23] Age UK . Social distancing, self isolation and shielding. Age UK, 2020, https://www.ageuk.org.uk/information-advice/coronavirus/coronavirus-guidance/social-distancing-and-self-isolation/

[24] HM Government. Caring for our future: reforming care and support. Victoria BC, Canada: Crown Publications, 2012.

[25] Dabelko-Schoeny H King S . In their own words: participants’ perceptions of the impact of adult day services. J Gerontol Soc Work 2010; 53: 176–192. 20094936 10.1080/01634370903475936

[26] Orellana K Manthorpe J Tinker A , et al. Day centres for older people: a systematically conducted scoping review of the literature about their benefits, purpose and how they are perceived. Ageing Soc 2020; 40: 73–104. 31798195 10.1017/s0144686x18000843 PMC6889849

[27] Anderson KA Geboy L Jarrott SE , et al. Developing a set of uniform outcome measures for adult day services. J Appl Gerontol 2020; 39: 670–676. 29900756 10.1177/0733464818782130

[28] Charlson M . Charlson Comorbidity Index. New York: Weill Cornell Medical College, 1987.

[29] Kaambwa B Gill L McCaffrey N , et al. An empirical comparison of the OPQoL-Brief, EQ5D3L and ASCOT in a community dwelling population of older people. Health Qual Life Outcomes 2015; 30: 164. 10.1186/s12955-015-0357-7 PMC4588872 26420314

[30] Euroqol. EQ-5D-3L about, https://euroqol.org/eq-5d-instruments/eq-5d-3l-about/ (2017, accessed 7 June 2019).

[31] De Jong Gierveld J Van Tilburg T . The De Jong Gierveld short scales for emotional and social loneliness: tested on data from 7 countries in the UN generations and gender surveys. Eur J Ageing 2010; 7: 121–130. 20730083 10.1007/s10433-010-0144-6 PMC2921057

[32] Orellana K Manthorpe J Tinker A . Day centres for older people – attender characteristics, access routes and outcomes of regular attendance: findings of exploratory mixed methods case study research. BMC Geriatr 2020; 20: 158. 32366223 10.1186/s12877-020-01529-4 PMC7197165

[33] Lunt C Dowrick C Lloyd-Williams M . What is the impact of day care on older people with long-term conditions: a systematic review. Health Soc Care Community. Epub ahead of print 17 December 2020. DOI: 10.1111/hsc.13245. 33332714

[34] Fried TR Tinetti ME Iannone L , et al. Health outcome prioritization as a tool for decision making among older persons with multiple chronic conditions. Arch Intern Med 2011; 171: 1854–1856. 21949032 10.1001/archinternmed.2011.424 PMC4036681

[35] Peplau LA Perlman D . Perspectives on loneliness. In: Peplau LA Perlman D (eds) Loneliness: a sourcebook of current theory, research and therapy. New York: John Wiley, 1982, pp. 1–18.

[36] Hagan R Taylor B Mallett J , et al. Older people, loss, and loneliness: the troublesome nature of increased contact with adult children illness. Crisis Loss 2020; 28: 275–293.

[37] Cornwell W . Social disconnectedness, perceived isolation, and health among older adults. J Health Soc Behav 2009; 50: 31–48. 19413133 10.1177/002214650905000103 PMC2756979

[38] De Bruin S Oosting S Tobi H . Comparing day care at green care farms and at regular day care facilities with regard to their effects on functional performance of community-dwelling older people with dementia. Dementia 2011; 11: 503–519.

